# Electrophysiological Indicators of the Age-Related Deterioration in the Sensitivity to Auditory Duration Deviance

**DOI:** 10.3389/fnagi.2016.00002

**Published:** 2016-01-22

**Authors:** Kamila Nowak, Anna Oron, Aneta Szymaszek, Miika Leminen, Risto Näätänen, Elzbieta Szelag

**Affiliations:** ^1^Laboratory of Neuropsychology, Nencki Institute of Experimental BiologyWarsaw, Poland; ^2^University of Social Sciences and HumanitiesWarsaw, Poland; ^3^Center of Functionally Integrative Neuroscience (CFIN), Aarhus University/Aarhus University HospitalAarhus, Denmark; ^4^Cognitive Brain Research Unit, Cognitive Science, Institute of Behavioural Sciences, University of HelsinkiHelsinki, Finland; ^5^Department of Psychology, University of TartuTartu, Estonia

**Keywords:** duration discrimination, mismatch negativity (MMN), P3a, aging, temporal information processing

## Abstract

The present study investigates age-related changes in duration discrimination in millisecond time domain. We tested young (*N* = 20, mean age = 24.5, *SD* = 2.97) and elderly (*N* = 20, mean age = 65.2, *SD* = 2.94) subjects using the mismatch negativity (MMN) paradigm. White-noise bursts of two different durations (50 and 10 ms) were presented in two oddball blocks. In one block (Increment Condition), the repetitive sequence of 10 ms standards was interspersed by occasional 50 ms deviants. In the Decrement Condition, the roles of the two stimuli were reversed. We analyzed the P1-N1 complex, MMN and P3a and found the effect of age for all these components. Moreover, the impact of stimulus presentation condition (increment/decrement) was observed for MMN and P3a. Our results confirmed the previous evidence for deteriorated duration discrimination in elderly people. Additionally, we found that this effect may be influenced by procedural factors.

## Introduction

The most pronounced symptom of cognitive aging is the deterioration of mental functions, like short-term memory, learning ability, attentional resources, language and motor activity. This is supported not only by everyday observations of elderly people, but also by numerous neuropsychological and psychophysical studies (e.g., Szelag et al., 2011 for a recent review). Cognitive functions mentioned in the foregoing are characterized by temporal dynamics at different time domains (milliseconds, seconds etc., e.g., Pöppel, 1994, 1997; Grondin, 2010; Rammsayer and Ulrich, 2011); consequently the efficiency of processing of time-related information exerts an influence on the human cognition at many different levels of complexity, from simple event perception to higher cognitive processes, such as planning or decision making. The relationship between temporal information processing (TIP) and other cognitive functions was investigated in many previous studies (e.g., Fitzgibbons and Gordon-Salant, 1994; Szelag et al., 2004, 2011; Kolodziejczyk and Szelag, 2008) which showed that especially the TIP in a time domain of some tens of milliseconds (millisecond timing) is crucial for the efficiency of human cognition. Additionally, the results of the previous studies confirmed that age-related deterioration of many cognitive functions in aging is accompanied by a decline of the TIP.

The TIP is commonly measured with two experimental paradigms: temporal order judgment (TOJ) and duration discrimination. Some similarities in the crucial time domain between these two paradigms can be observed. The effectiveness the TOJ is reflected by the temporal order threshold which is the minimum length of the stimulus-onset-asynchrony (SOA) required to report correctly the order of two events presented in a rapid succession and corresponds to a temporal gap of some tens of milliseconds, on average from 20–90 ms (Lapid et al., 2008; Szymaszek et al., 2009). On the other hand, duration discrimination refers to the ability to discriminate the smallest possible difference in duration (difference limen) which usually has a duration similar to that reported for TOJ, thus of ca. 30–60 ms (e.g., Lapid et al., 2008). Moreover, both the TOJ and duration discrimination decline with age (e.g., Fitzgibbons and Gordon-Salant, 1994; Kolodziejczyk and Szelag, 2008; Szymaszek et al., 2009; Kumar and Sangamanatha, 2011).

In the present study, we investigated age-related decline in duration comparison on a millisecond level using the passive oddball task to elicit the mismatch negativity component (MMN; Näätänen et al., 1978). The MMN is a fronto-central, negative event-related potential (ERP) that usually peaks ca. 100–200 ms from the onset of a detectable change (“deviant”) in a repetitive sequence of stimuli (“standards”). The MMN reflects the operation of a pre-attentive memory-based comparison mechanism. As standards form a memory trace in the central auditory system, a rare different stimulus (“deviant”) elicits the MMN. Compared to psychophysical methods, the MMN procedure has an important advantage as it requires no active participant response. Thus, it seems to be free from overlap by other factors that could influence the measurements such as those associated with decision making, attentional focus, verbal output or movement control that are often required in psychophysical methods (for a review, see Picton et al., 2000; Kujala et al., 2007).

Several studies have shown that the amplitude and peak latency of the MMN correlate both with the task difficulty and the complexity of the stimulus presented. The amplitude increases and peak latency decreases when the difference between deviants and standards becomes more evident (Näätänen et al., 1978, 2007; Picton et al., 2000; Bishop, 2007; Kujala et al., 2007). The relationship between the MMN and psychophysical measurements for the accuracy of sound-duration discrimination was demonstrated by Amenedo and Escera (2000). However, there is also some evidence showing no correlation between MMN amplitude and the deviance magnitude (Horváth et al., 2008).

Previous studies on the MMN in aging usually showed a reduced amplitude and prolonged latency in elderly people (ca. 70 years) in comparison to those in the younger ones (ca. 25 years). This result was interpreted as indicating age-related deficits in the encoding of sound properties in auditory sensory memory (e.g., Gaeta et al., 1998; Cooper et al., 2006). It may also indicate the poorer performance of comparator or time-perception mechanisms which become less sensitive with an increasing age for small differences between deviants and standards (Gaeta et al., 1998). Other studies (Alain and Woods, 1999; Kisley et al., 2005) also found that a diminished MMN amplitude may partly result from an enhanced N1 amplitude in elderly people to task-irrelevant stimulation (for a review, see Tomé et al., 2015 ). This was interpreted in terms of declined inhibitory control related to age-related dysfunction of the prefrontal cortex (Chao and Knight, 1997; Kisley et al., 2005; for details, see “Discussion” Section).

Although several studies were focused on age-related changes in the MMN response, only a few dealt with differences in the MMN in duration discrimination. For example, Joutsiniemi et al. (1998) performed an MMN experiment using deviant stimuli that were either 25 or 50 ms shorter than the standard of 75 ms duration. Healthy subjects aged from 9–84 years participated in this study which was focused on the replicability of the MMN. Their main finding was that the 25 ms deviants elicited less replicable and more variable MMN responses than did the 50 ms deviants. It was also shown that the MMN amplitude decreased with age, which supports the notion of a declined TIP in aging. In another study, Cooper et al. (2006), using frequency or duration deviants, found that the MMN amplitude was smaller and the latency longer in the elderly for both kinds of deviants. This result was interpreted in terms of declined auditory sensory memory in elderly people. Both these studies have some limitations as only one duration discrimination condition was applied, i.e., either duration decrement (Joutsiniemi et al., 1998) or duration increment (Cooper et al., 2006).

In addition, the difference waves were obtained by subtracting the ERPs for standards from those for deviants of different durations. Such a method did not allow to separate the effect of duration discrimination from neural responses to physical characteristics of the stimuli used (i.e., differences in stimulus duration, Ostroff et al., 2003). As shown by Peter et al. (2010) and see also Jacobsen and Schröger (2003), the way of obtaining the difference wave has an impact on the MMN amplitude and may affect the results obtained. Therefore, in the present study, the difference wave was calculated using the “same-stimulus method” based on a subtraction of the ERPs to identical stimuli presented as standard in one block and as deviant in another block. Such a procedure allows one to extract the genuine MMN response to duration deviance (Jacobsen and Schröger, 2003; Peter et al., 2010). Moreover, to investigate the possible contribution of the N1 to the MMN response we analyzed also the peak-to-peak amplitude of the N1 (P1/N1 Complex) in response to standards (Woods, 1995). Finally, the observed P3a component, related to engagement of attention, was analyzed (e.g., Polich, 2007).

To sum up, on a basis of previous studies, the present study is aimed at determining age-related changes in electrophysiological response in auditory duration discrimination task using both duration increment (deviant longer than standard) and decrement (deviant shorter than standard) conditions.

## Procedure

### Ethical Approval

The study protocol was approved by the Ethic Comission at the University of Social Science and Humanities (permission no 3/II/11-12). The study was conducted according to the Helsinki Declaration. The written informed consent from each participant was obtained prior to the testing.

### Participants

Forty participants were recruited for this study. They were classified into two age groups: 20–29 years old (the young group; age: $\bar{x}$ ± *SD* = 24.5 years ± 2.97, *n* = 20) and 60–69 years old (the elderly group; age: $\bar{x}$ ± *SD* = 65.2 years ± 2.94, *n* = 20). These two groups were balanced with respect to gender (a half males/a half females in each age group).

All subjects were right-handed (Edinburgh Handedness Inventory; Oldfield, 1971), with no history of neurological or psychiatric diseases and had a normal hearing level, verified by the screening audiometry (Audiometer AS 208). All the subjects showed the normal hearing level (ANSI, 2004) for frequency range from 250–3000 Hz which covered the main spectrum of the stimuli presented in this study.

Additionally, the elderly participants were tested with the Mini-Mental State Examination (MMSE) to screen for mental deterioration. An inclusion criterion was a score above 27 points. All subjects achieved the maximum score (total score = 30), except for one female who achieved 29 points.

### Materials and Methods

Stimuli were two white-noise bursts of 50 and 10 ms duration with instantaneous rise/fall time. They were delivered using Presentation Software version 14.9 (Neurobehavioral Systems Inc.) binaurally with loudness of 80 dB (SPL) *via* E·A·RTone^®^ 5A Insert Earphone headphones, plugged into the ear canal. Two oddball blocks were created. In one block, a sequence of long-standards (50 ms) was occasionally interspersed with short, 10 ms deviants (“Decrement Condition”). In the other block (“Increment Condition”), 10 ms sounds were delivered as standards (short-standards) and 50 ms sounds as deviants (long-deviants). Each of the two blocks consisted of 400 stimuli which were presented with an offset-to-onset ISI of 500 ms. The standard to deviant ratio was 80 to 20%. In each block, 10 standards were presented in the beginning and the distribution of the deviants was quasi-randomized, i.e., maximally two consecutive deviants could be presented in a row. The block order was randomized between the subjects. During the experiment, participants were asked to watch self-selected, silent, subtitled video and ignore any auditory stimulation.

#### Electrophysiological Data Recording and the ERP Analysis

The FCz-referenced electroencephalogram (EEG) was recorded from 32-channel electrode cap (EasyCap, Germany) with Ag/AgCl active electrodes (ActiCAP, Brain Products, Germany) placed according to the 10–20 system, by BrainAmp EEG amplifier (Brain Products, Germany; bandpass filter of 0.1–100 Hz and sampling rate of 1000 Hz). The electrode contact impedances were kept below 10 kΩ. The EEG was recorded by BrainVision Recorder© v.1.10 software (Brain Products, Germany).

Offline signal processing was performed with Brain Vision Analyzer v.2.0 (Brain Products, Germany). The data were re-referenced to the average of mastoids and filtered with passband of 1–30 Hz (zero-phase Butterworth with 24 dB/octave). Eye blinks and horizontal eye movements were corrected from the continuous signal using Independent Component Analysis (ICA). Individual ERP data from each participant were inspected visually and maximally 2–3 clearly eye-related components were removed. Epochs of 500 ms (including a 100 ms pre-stimulus period) were created with time 0 marking the stimulus onset. After baseline correction (from −100 to 0 ms), trials with a charge of EEG exceeding ±90 μV amplitude were excluded from the analysis. Epochs were then separately averaged for the four stimulus types: long-standards, long-deviants, short-standards, short-deviants. Difference waves were obtained by subtracting physically identical standards from deviants (“same-stimulus method”), i.e., “long standards” from “long deviants” and “short standards” from “short deviants” (Jacobsen and Schröger, 2003).

**Figure 1:**
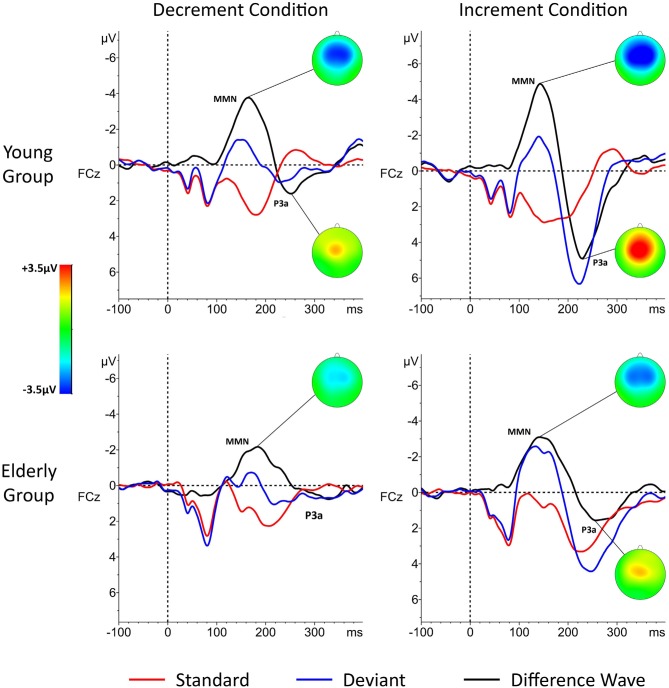
**Grand-averages of event-related potential (ERP) responses in young and elderly participants in the “Decrement Condition” (10 ms deviant; left) and in “Increment Condition” (50 ms deviant; right).** Difference waves (black lines) were obtained for each condition by subtracting physically identical stimuli. Time “zero” indicated stimulus onset. The P1-N1 Complex (the P1-N1 peak-to-peak amplitude) was analyzed for standard stimuli only (red line).

**Figure 2:**
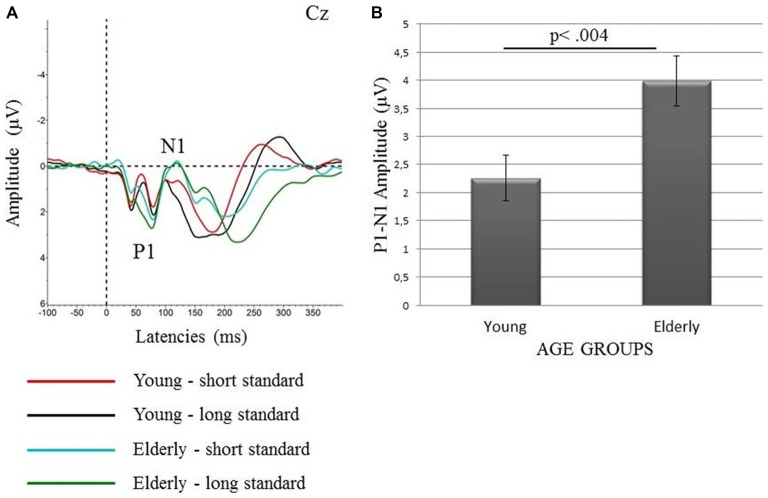
**Grand-averages of the ERP waveforms to long and short standards (A) and the mean values of the peak-to-peak amplitudes of the P1-N1 complex (with SEM) for both standards (50 and 10 ms) together in the young and elderly participants (B)**.

After the visual inspection of the averaged ERP obtained for both standards and deviants, as well as for the calculated differences, we performed three analyses as follows:

1. The P1-N1 peak-to-peak amplitudes were analyzed for the response to standard stimuli, both long and short ones on the central electrode (Cz). The P1 component was identified as the most positive value at a time window of 50–100 ms after stimulus onset. The N1 was identified as the most negative value in a time window of 70–170 ms after stimulus onset. Due to the passive oddball paradigm as well as the rapid stimuli presentation, the amplitude of the N1 component was rather low (see Figures 1, 2A) which is consistent with previous findings (Hillyard et al., 1973; Näätänen and Picton, 1987; Polich et al., 1988).
2. The MMN, analyzed from the difference waves, was identified within a time window from 110–230 ms, according to the grand averages of the MMN responses on FCz (Figure 1). EEG data from nine electrodes: frontal (F3, Fz, F4), fronto-central (FC1, FCz, FC2) and central (C3, Cz, C4) were included in the analysis. The electrodes were pooled in two spatial dimensions: “anterior-posterior” and “lateral” in order to look for spatial differences in the MMN topography. For the “anterior-posterior” dimension, three lines of pooled-electrodes were obtained: “frontal” (F3, Fz, F4), “fronto-central” (FC1, FCz, FC2), and “central” lines (C3, Cz, C4). The pooling of the electrodes in the “lateral” dimension also resulted in three lines of pooled electrodes: “left” (F3, FC1, C3), “midline” (Fz, FCz, Cz), and “right” (F4, FC2, C4).
  The peak latencies for each participant were analyzed at FCz because of the highest peak and thus the highest signal-to-noise ratio at this electrode.
3. Additionally, we observed a clear P3a component which, according to the previous studies, reflects the stimulus-driven reorienting of attention (e.g., Escera et al., 1998, 2001; Goldstein et al., 2002). The P3a usually follows the MMN, at least when the difference between standard and deviant becomes intrusive (Figure 1). A visual inspection revealed that it was present in both age groups, however in elderly it was not clear in the Decrement Condition (see Figure 1). To investigate age-related differences for this component, we analyzed the most positive values in a time window 200–350 ms after stimulus onset. All analyses for P3a were performed on pooled electrodes in both dimensions, similarly as for MMN (see above).

#### Statistical Analyses

##### The P1-N1 complex

Mean values of the peak-to-peak amplitudes obtained in each individual were submitted to two-factor repeated measures analysis of variance (ANOVA 1) with “Age” (young, elderly) as between-subject factor and “Condition” (Increment, Decrement) as a within-subject factor.

##### MMN component

The mean amplitudes and latencies of the MMN obtained in each participant were separately considered. For the amplitudes, two 3-factor ANOVAs (2 and 3) were performed for each pooling dimension. For the anterior-posterior dimension, ANOVA 2 was conducted with “Age” (young, elderly) as a between-subject factor and “Electrode Region” (frontal, fronto-central and central) and “Condition” (Increment, Decrement) as within subject factors. For the lateral dimension, ANOVA 3 was conducted with the same between- and within-subject factors, as those used in ANOVA 2. The only difference between ANOVA 2 and 3 was in the factor “Electrode Region”: anterior-posterior (ANOVA 2) or Lateral (ANOVA 3).

For the peak latencies, a two-factor ANOVA 4 with “Age” (young, elderly) as a between-subject variable and “Condition” (Increment, Decrement) as a within-subject variable was conducted on the FCz electrode. After these analyses, the Bonferroni *post hoc* test was applied to study further the main effects and interactions observed.

##### The P3a component

The mean amplitudes from the P3a time window were submitted to a three-factor ANOVA 5 for the anterior-posterior pooling dimension and to ANOVA 6 for the lateral one. Each of these ANOVAs included the same between- and within-subject factors, as the analyses performed for the MMN amplitudes (see above).

## Results

### P1-N1 Complex

ANOVA 1 only revealed the main effect of “Age”, *F*_1,38_ = 9.45, *P* < 0.004, with higher peak-to-peak amplitudes in the elderly than in the young group. This result is presented in Figure 2. To determine the contribution of the P1 and N1 components to this effect, we performed additional *t*-tests for the amplitudes of these two components. Although the difference between the amplitudes of the P1 and N1 peaks was visible in grand averages (Figure 2), the statistical analysis revealed that these differences were nonsignificant.

### The MMN Component

#### Amplitudes

**Figure 3:**
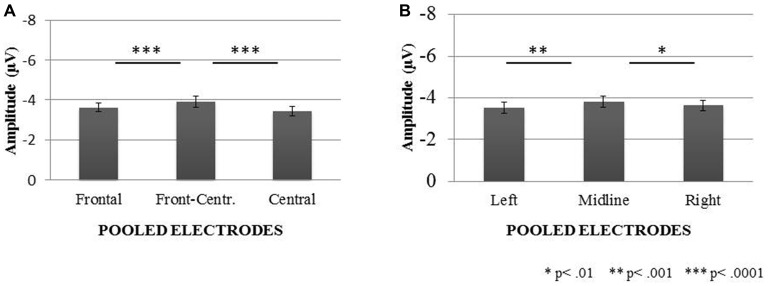
**Mismatch negativity (MMN) peak amplitudes (with SEM) registered on electrodes pooled in two dimensions: anterior-posterior (A) and lateral (B)**.

ANOVA 2 (anterior-posterior pooling) showed main effects of: “Age”, *F*_1,38_ = 5.92, *P* < 0.02, “Condition”, *F*_1,38_ = 18.19, *P* < 0.0001, and “Electrode Region”, *F*_2,76_ = 16.41, *P* < 0.0001. The same main effects were significant in ANOVA 3 (lateral pooling): “Age”, *F*_1,38_ = 5.92, *P* < 0.02, “Condition”, *F*_1,38_ = 18.19, *P* < 0.0001, and “Electrode Region”, *F*_2,76_ = 8.77, *P* < 0.001. In both these ANOVAs, the MMN peak amplitudes were lower in the elderly than in the young group, as well as in the Decrement Condition than in the Increment Condition. In ANOVA 2, the largest MMN amplitudes were registered on the fronto-central electrodes in comparison to the frontal (*P* < 0.0001) and central electrodes (*P* < 0.0001). In ANOVA 3, the MMN peak amplitudes registered on the midline electrodes were significantly higher than those registered on the left (*P* < 0.001) and right (*P* < 0.01) electrode lines. The other differences in ANOVAs 2 and 3 were nonsignificant. These relationships are displayed in Figure 3.

#### Latencies

**Figure 4:**
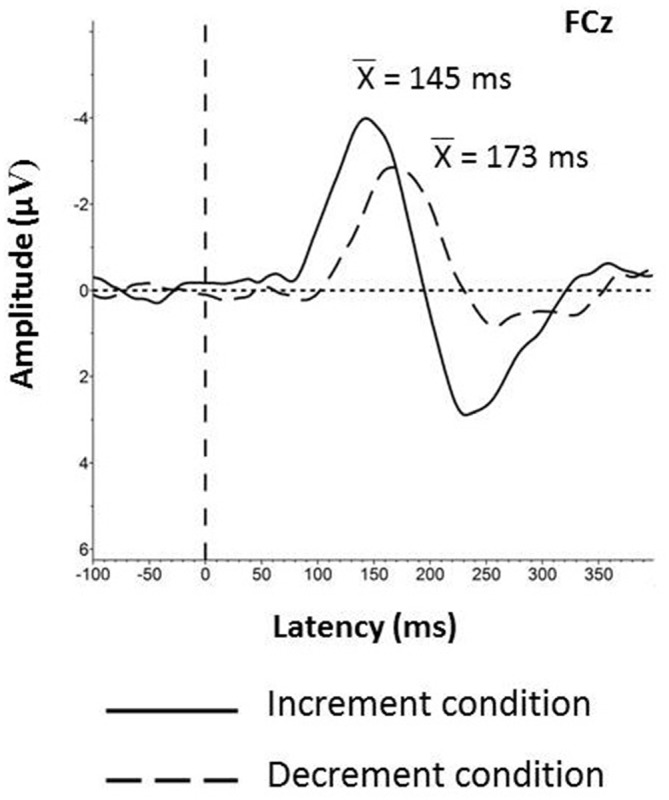
**Grand averages obtained for all participants with the mean values of the MMN peak latencies registered on the FCz electrode in the Increment and Decrement Conditions.** Latency 0 represents stimulus onset.

The results of ANOVA 4 conducted on the peak latencies revealed the main effect of “Condition”, *F*_1,38_ = 46.57, *P* < 0.0001. Generally, the MMN peak latencies were shorter in the Increment than in the Decrement Condition for both age groups (Figure 4).

### P3a Component

For the anterior-posterior pooling ANOVA 5 revealed a main effect of “Age”, *F*_1,38_ = 15.52, *P* < 0.0001, “Condition”, *F*_1,38_ = 29.9, *P* < 0.0001, “Electrode Region”, *F*_2,78_ = 16.88, *P* < 0.0001. These main effects were modified by two interactions: “Age × Condition”, *F*_2,76_ = 5.78, *P* < 0.03 and “Condition × Electrode Region”, *F*_2,76_ = 9.95, *P* < 0.0001.

Generally, the P3a amplitudes were larger in the Increment Condition than in the Decrement Condition. The interaction “Condition × Electrode Region” resulted from significant amplitude differences but only in the Increment Condition, with significantly larger amplitudes on the fronto-central electrode line (3.77 μV, ±2.42 μV) than on frontal (3.11 μV, ±1.98 μV) and central (3.48 μV, ±2.15 μV) ones. Additionally, the amplitudes on the central electrodes were significantly larger than those on the frontal electrodes (*P* < 0.02).

**Figure 5:**
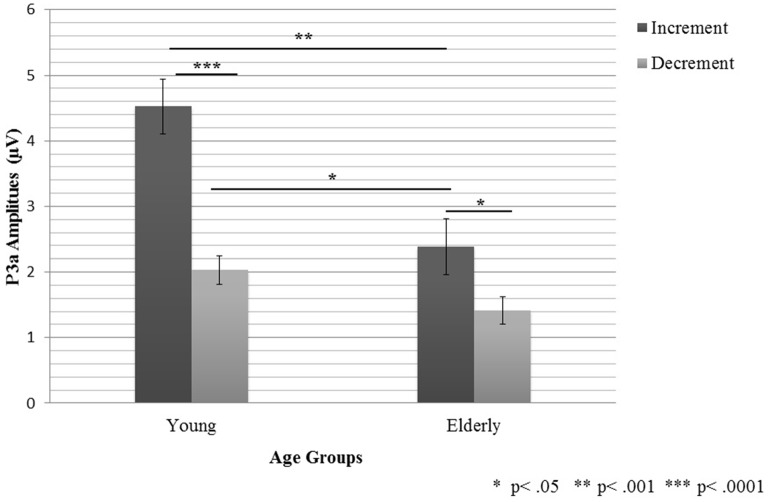
**The P3a peak amplitudes (with SEM) registered in young and elderly participants in the two experimental conditions**.

Although the P3a amplitudes were generally larger in the young than elderly subjects, this effect was much stronger in the Increment (*P* < 0.001) than in the Decrement Condition (*P* < 0.05). Moreover, in the elderly, the difference between conditions was smaller (*P* < 0.04) than that in the young subjects (*P* < 0.0001). This effect is shown in the Figure 5.

For the lateral pooling (ANOVA 6), three main effects were obtained: “Age”, *F*_1,38_ = 15.52, *P* < 0.001, “Condition”, *F*_1,38_ = 29.9, *P* < 0.001 and “Electrode Region”, *F*_2,76_ = 19.84, *P* < 0.001. These effects were modified by two-factor interactions: “Age × Condition”, *F*_1,36_ = 5.78, *P* < 0.03, “Age × Electrode Region”, *F*_2,76_ = 8.65, *P* < 0.001 and “Condition × Electrode Region”, *F*_2,76_ = 7.52, *P* < 0.001. In general, these relationships reflect lower amplitudes in the elderly than in the young subjects, as well as in the Decrement than in the Increment Condition. Moreover, the differences between the conditions were larger in the young (*P* < 0.0001) than in the elderly group (*P* < 0.04), similarly as in ANOVA 5. Additionally, only in the young group, differences between the electrode regions were observed, with the highest amplitudes on the midline electrodes.

### Summary of Results

Two important effects were revealed in the present study: those for the age and the stimulus-presentation condition. The age effect was indicated for all the components analyzed (P1-N1 Complex, MMN, and P3a). For the P1-N1 complex, larger peak-to-peak amplitudes were observed in the elderly than young participants (Figure 2). Moreover, the MMN amplitudes were reduced in the elderly people, but for the peak latencies no age effect was found (Figures 3, 4). The P3a component was more pronounced in the young than in elderly group (Figure 5). Additionally, the P3a differences between the pooled electrode lines were found in the young group only (Figure 5).

The other main effect reported here was the stimulus-presentation condition. It was observed for the MMN and P3a components, but not for the P1-N1 Complex. The MMN component was elicited earlier and with larger amplitudes in the Increment than Decrement Condition (Figure 4). The similar relation was found for the P3a amplitudes in the young group. Differences between the electrode lines within each condition were observed in the young group only. The largest amplitudes were found on the fronto-central electrodes for the anterior-posterior pooling and on the midline electrodes for the lateral pooling direction (Figure 5). To sum up, the age of the participants had an impact on all components registered (P1-N1 Complex, MMN, P3a). However, this effect was modified by the stimulus-presentation condition.

## Discussion

The present study was focused on age-related differences in auditory duration discrimination in millisecond time domain. In comparison to the previous MMN studies on duration discrimination in aging (Joutsiniemi et al., 1998; Cooper et al., 2006), our study has some methodological advantages. The present difference waves (“same-stimulus” method) allowed us to extract the brain response related only to the detection of duration deviance (both for duration increments and decrements).

### The Effect of Age

In the present study, the age effect was revealed for all ERP components analyzed, i.e., the P1-N1 complex, the MMN and the P3a.

For the P1-N1 complex, the impact of age was observed for the peak-to-peak amplitudes, with increasing amplitudes in the elderly in comparison to the young subjects (Figure 2), consistent with previous literature results (e.g., Alain and Woods, 1999; Kisley et al., 2005) indicating a higher N1 amplitude in elderly people to irrelevant auditory stimuli. This effect may be related to the impaired inhibitory control of sensory input associated with declined executive function with the advancing age. Kisley et al. (2005) suggested that the larger N1 amplitudes manifested in the elderly may result from the enhanced primary auditory processing of task-irrelevant stimulation. Such an enhancement was previously found in both older adults and patients with lesions to the prefrontal cortex. Thus, the results obtained by Kisley et al. (2005) may also support the hypothesis of an age-related dysfunction of the prefrontal cortex and executive control. Referring to these studies, we hypothesize that the age effect for the P1-N1 complex may be associated with changes in the prefrontal cortex which seem to cause the failure to suppress irrelevant stimuli (see Chao and Knight, 1997).

Regarding the MMN, the present age-related decrease of the amplitude reported here is consistent with the results obtained by Joutsiniemi et al. (1998), as well as by Cooper et al. (2006). Using both duration Increment and Decrement Conditions, as well as the “same-stimulus method”, we confirmed that the MMN amplitude is declined with age. We also showed that the effect of the age on electrophysiological responses may be modulated by the stimulus-presentation condition (see below). It seems that this decline may result from deficits in auditory sensory memory, as previously shown by Cooper et al. (2006). On the other hand, lower MMN amplitudes could also be explained by deficient TIP in which prefrontal cortex may be involved (Lewandowska et al., 2010). According to Kraus and Anderson (2013), the decreased precision in the processing of the acoustic stimuli may also result from the neural slowing in older adults.

Age-related differences for the P3a complement the MMN results, suggesting that elderly people exhibit a reduced ability of duration discrimination. In our study, the P3a was observed in both conditions in young participants, whereas in the elderly, it was visible only in the Increment Condition. According to the existing literature (for a review, see Polich, 2007), the decreased P3a amplitudes and increased peaks-latencies might reflect a frontal lobe dysfunction in the elderly people. The diminished P3a amplitudes found in both previous reports and in our study may reflect age-related changes within this brain area which may contribute to age-related deficits in duration discrimination.

### The Effect of the Direction of the Duration Change: Increment vs. Decrement

We observed that the stimulus-presentation condition had an impact on both the MMN and P3a components. In general, the MMN amplitudes were higher in the Increment than Decrement Condition, despite the constant 40 ms difference between the standards and deviants in these reversed conditions.

As mentioned in the “Introduction” Section, the previous studies on duration discrimination revealed equivocal relation between these two conditions. Such an ambiguity of existing results may be caused by several factors, such as variety of applied stimuli (e.g., tones, white noises, chords; Takegata et al., 2008) or data analysis method (e.g., Picton et al., 2000; Bishop, 2007; Näätänen et al., 2007; Peter et al., 2010).

To our knowledge, there are only a few papers considering the effect of deviance direction on the MMN (increment or decrement). Amenedo and Escera (2000) and Peter et al. (2010) found no differences between the MMN amplitudes for duration decrement and increment. On the other hand, Colin et al. (2009) showed that the MMN was elicited earlier and with larger amplitude for duration decrement (short deviants) than for duration increment (long deviants). Moreover, Okazaki et al. (2006), using an animal model (studying guinea pigs), obtained longer peak latencies in the Increment than Decrement Condition, especially for longer duration differences.

Our results are inconsistent with the previous studies as we found larger MMN amplitudes and shorter peak latencies in the Increment than Decrement Condition. This result may be explained in terms of the specifity of stimuli applied here. As reported by Takegata et al. (2008), sound type may have an effect on the increment and decrement MMN. These authors found that for white-noise stimuli (presented also in our study), MMN peak was elicited earlier and with a larger amplitude in the Increment than in Decrement Condition. Our study confirmed this result.

Although the present results showed that stimulus-presentation condition had an impact on the ERP amplitudes, they did not fully contradict the finding by Amenedo and Escera (2000) who found that the magnitude of the MMN was proportional to a difference between standards and deviants. In their study the deviants differed from the standards by 10, 20, 30, 40 or 60% in both directions. It should be noted that in our study, despite the same absolute duration difference in both conditions (40 ms), the relative difference varied between Increment and Decrement Condition. In Increment Condition standard was five times longer (500%) than the deviant (50 ms). On the other hand, in Decrement Condition such relative difference was 80%, because the deviant comprised only 20% of the standard. This may be reflected in the MMN response, as its amplitude correlated positively with the contrast size (e.g., Näätänen et al., 1989; Amenedo and Escera, 2000). The higher contrast between the exposed stimuli (Increment Condition) may create an easier perceptual situation, eliciting higher and earlier MMN in both groups.

Another explanation for the effect of stimulus presentation condition may be rooted in psychophysical studies on loudness summation. Accordingly, two stimuli of equal objective loudness, but differing in duration are subjectively perceived as differing in loudness, with longer stimulus being perceived as more intensive one (Zwislocki, 1969). The application of same-stimulus method allows the MMN calculation for the sounds with physically identical intensity. However, it is possible that the subjectively perceived loudness overlapped with the perception of duration deviance (see above) in both conditions. It is possible that in the Decrement Condition the listeners might become more adapted to the subjectively louder sound, while in the other condition they adapted to the less intensive one. This phenomenon could also explain the shorter latency for Increment than for Decrement Condition.

The results obtained for the P3a are similar to those observed for the MMN. The higher P3a amplitudes in the Increment than Decrement Condition in the young group confirm that the durations difference was rather easily detectable in the Increment Condition. A similar effect of condition was also present in elderly participants, however, in this group, the P3a was observed in the Increment Condition only. Such a result may suggest that the relative difference between standard and deviant in the Decrement Condition, might be not sufficient to elicit P3a, related to attentional shift to an intrusive stimulus, in the elderly.

To sum up, the present study confirms the results obtained in the previous MMN research focused on duration discrimination in aging, but provides also some insight into procedural factors affecting the results. Considering age-related differences in electrophysiological response to duration deviance, the data presented here reflect the fact that the sensitivity to duration deviance declines with age which was evidenced by both the MMN and P3a data. Consequently, we confirmed the previous findings on aging with a methodologically improved procedure, and added some new insight on TIP as a function of aging. We also revealed that the age-related differences may be modified by the relative magnitude of the duration change.

## Conflict of Interest Statement

The authors declare that the research was conducted in the absence of any commercial or financial relationships that could be construed as a potential conflict of interest.
